# Chaos Detection in Noisy Signals Using Refined Signal Representations

**DOI:** 10.3390/s26175682

**Published:** 2026-09-07

**Authors:** Katarzyna Harężlak, Dariusz R. Augustyn, Henryk Josiński, Adam Świtoński, Paweł Kasprowski, Agnieszka Szczęsna

**Affiliations:** 1 Silesian University of Technology, Faculty of Automatic Control, Electronics and Computer Science, Department of Applied Informatics, Akademicka 16, 44-100 Gliwice, Poland; katarzyna.harezlak@polsl.pl (K.H.); dariusz.augustyn@polsl.pl (D.R.A.); pawel.kasprowski@polsl.pl (P.K.); 2 Silesian University of Technology, Faculty of Automatic Control, Electronics and Computer Science, Department of Computer Graphics, Vision and Digital Systems, Akademicka 16, 44-100 Gliwice, Poland; henryk.josinski@polsl.pl (H.J.); adam.switonski@polsl.pl (A.Ś.)

**Keywords:** nonlinear dynamics, time series, phase portrait, chaos detection, noise

## Abstract

Discovering the characteristics of nonlinear dynamical systems is an important topic in many fields. When the governing equations are known, such analysis is straightforward; otherwise, as in the case of biological data, alternative approaches are required. This paper presents one such approach and is a continuation of previous studies on differentiating chaotic and non-chaotic behavior using machine learning and synthetic datasets generated from well-known dynamical systems. These datasets were used to extract refined representations, defined as groups of data with similar initial conditions. The method relies on phase-space reconstruction and data clustering. The refined representations were used for the classification of system dynamics using a Long Short-Term Memory (LSTM) network. The model was trained on both noise-free and noise-contaminated data and evaluated on noisy test sets from different dynamical systems. Results obtained for the refined representations were compared with those obtained for the original ones. The experiments showed that models trained on the refined representation generally achieved better classification performance, particularly under moderate-to-high noise conditions. However, this advantage was not consistent across all noise conditions, indicating that the effectiveness of the refined representation depends on both the type and the level of noise, as well as on the noise characteristics of the training data.

## 1. Introduction

Deterministic chaos, characterized by extreme sensitivity to infinitesimally small perturbations, is a prevalent phenomenon in biological signals. These tiny changes in initial conditions are amplified and have such a substantial impact on the final state that the behavior of such a system cannot be predicted over a longer time horizon [1]. This sensitivity implies that seemingly random variations may stem from underlying deterministic processes. For example, chaotic behaviors observed in human movement pertain to complex, seemingly random yet deterministic motion patterns resulting from the nonlinear dynamics of the neuromuscular system. Such dynamics enable adaptability and responsiveness to varying environmental conditions. For instance, healthy flexibilityin human gait refers to the body’s ability to adapt to changes in the ground surface, speed, and environmental conditions while maintaining stability and minimizing the risk of falls [2].

Previous studies consistently demonstrated that noise contamination significantly degrades the performance of time-series classification systems. A consistent monotonic decrease in classification accuracy of simulated signals with increasing noise level was observed, where six state-of-the-art classifiers achieve approximately 64.61–100% accuracy under low-noise conditions (with noise standard deviation equals 5), which decreases to around 5.82–63.13% under high-noise (with noise standard deviation equals 50) regimes depending on the classifier, and number of classes [3]. Also, improperly modeled noise adversely affects the adequacy of time-series models and the reliability of forecasts, leading to distorted statistical characteristics extracted from temporal data [4]. The study [5] investigates the ability of deep learning models to distinguish deterministic chaotic dynamics from stochastic noise. Classification performance deteriorated as the level of observational noise increased, corresponding to a decrease in the effective signal-to-noise ratio (SNR). Deep learning methods, particularly CNN-based architectures, demonstrate improved robustness to noise, especially in biomedical signal classification, compared to traditional handcrafted feature approaches, owing to their ability to automatically learn hierarchical spatial–temporal representations from raw or transformed signals. However, their performance still deteriorates under low signal-to-noise ratio conditions and in noisy real-world clinical environments [6,7]. Measurement noise can obscure class-specific temporal patterns and reduce classification accuracy, motivating the use of noise-tolerant representations and classifiers that preserve discriminative information under signal perturbations [8,9].

Noise and intrinsic variability are inherent components of biological systems and may contribute to their flexibility, plasticity, information transfer, and ability to respond to the internal and external perturbations [10] which are typically generated by complex systems whose governing equations are unknown. Methods for detecting chaos from empirical measurements are crucial in understanding the functioning of these systems. However, noise is an inherent part of biomedical signal acquisition arising from various factors. Patient movements and physiological activities can generate internal noise, affecting signal quality. External environmental factors such as electromagnetic fields and nearby electronic devices can introduce noise into signals, as can imperfections in the measuring or recording process and devices, including sensor inaccuracies (e.g., electrodes and preamplifier) and signal processing errors [11,12]. Traditional methods for identifying chaos are susceptible to measurement noise and often fail in typical edge cases, making chaos detection challenging [13]. Therefore, recognizing chaotic patterns and distinguishing deterministic chaos from stochastic noise helps understand the complex system dynamics, which is important for further signal processing and analysis, ensuring the application of appropriate analytical methods, algorithms, and measures.

To address this challenge, mathematical dynamical systems with known chaotic and non-chaotic regimes were employed as a source of ground-truth data. To better mimic the conditions encountered in biological recordings, these systems were investigated under different noise levels. We hypothesize that models trained on such data can learn robust dynamical features and subsequently be used to assess the presence of chaos in biological signals for which no ground truth is available.

The starting point for the study described in this paper was a collection of datasets developed for machine learning methods aimed at classifying the examined signals as chaotic or non-chaotic [14,15]. One of these datasets, referred to as *Refined* dataset [15], consist of groups of signals generated under very similar (though not identical) initial conditions. They were created by clustering signals produced by models of well-known chaotic and non-chaotic dynamical systems, stored in the *Original* dataset [14].

This study evaluated the performance of refined signals (included in the *Refined* dataset) in the classification of chaotic and non-chaotic behaviour under varying noise conditions. The results were compared with those obtained using the original signal representation (the *Original* dataset) and a dataset combining original and refined signals (the *Augmented* dataset). A recurrent LSTM network was used in three experimental scenarios, covering both noise-free and noise-contaminated training conditions.

The contribution of this study is a systematic evaluation of the robustness of the previously proposed refined representation under varying levels of signal degradation and different training conditions. In particular, the study investigates its performance when models trained on clean signals are applied to noisy data, as well as when noise is present during the training process. The results demonstrate that the effectiveness of the refined representation depends on both the degree of signal degradation and the characteristics of the training data. By identifying the conditions under which the refined representation provides the greatest benefits, this work extends previous research and offers a more comprehensive understanding of its robustness.

## 2. State of the Art

The guarantee of obtaining data with specific properties for machine learning needs is the use of nonlinear models of dynamic systems, whose properties are well-known and well-described, because deterministic chaos occurs only for specific ranges of model parameter values. Professor Sprott’s book “Chaos and Time-Series Analysis” [16] describes a number of such systems, also providing the corresponding values of the largest Lyapunov exponent, which confirm the extremely strong sensitivity of the system to initial conditions.

Deep learning methods are increasingly used for classifying chaotic behavior in signals because they can automatically learn complex nonlinear temporal representations directly from raw time-series data without relying on manually engineered features. In contrast to traditional feature-based approaches, deep neural networks are better able to capture hidden deterministic structures and enhance classification robustness for complex nonlinear time-series data [17].

The authors of the paper [18] considered two discrete dynamical systems: the logistic map and the sine-circle map. The study aimed to find a neural network that can learn the features characterising chaotic signals of the logistic map and generalise to signals generated by the sine-circle map. The findings of this exploration indicate that a convolutional neural network without batch normalisation layers outperforms state-of-the-art neural networks for time-series classification and is able to generalise and classify time series as chaotic or not with high accuracy. The main disadvantage of the study is that a limited group of systems was taken into account. In [19], the objective was to classify different types of motion—namely chaotic, librational, or rotational—by analyzing samples extracted from time series generated by simple Hamiltonian systems.

The study described in [20] utilized deep learning techniques to classify various chaotic systems based on graphical representations of their time series. A dedicated dataset containing time-series images from the Chen and Rössler systems was generated and produced under varying parameter values, initial conditions, time steps, and durations. Classification was then conducted using transfer learning approaches, achieving high accuracy. The applied models include SqueezeNet, VGG-19, AlexNet, ResNet-50, ResNet-101, DenseNet-201, ShuffleNet, and GoogLeNet. The results demonstrate that it is possible to identify a chaotic system based solely on the visual appearance of its time series.

The study [21] presents a method for accurately estimating the Largest Lyapunov Exponent (LLE) from noisy data, based on training deep learning models on synthetically generated trajectories. The authors explored deep learning models designed to estimate the LLE, which is often used for classifying a system as chaotic, from tree trajectories for systems given by the recurrence relations. The study showed strong classification abilities even for relatively shallow input trees. A similar approach was utilized in [22], where the LLE was also estimated using deep neural networks in discrete dynamical systems based on observable trajectories within an extended state space. The proposed method was validated through simulations involving various system topologies and attractor complexities.

Another similar challenge to chaos evidence prediction is system identification. In this case, the dynamical models described by state equations are discriminated regardless of their initial conditions or even parameters. The classification takes into account the observation data, which are output signals or state variables. In [23], baseline Lorenz, Chen, and Rössler systems are classified using machine learning with 18 sub-methods based on Naive Bayes, Support Vector Machines, K-Nearest Neighbors, and Decision Tree techniques. In [24], multimodal deep neural networks combining information provided by recurrence plots and spectrograms are proposed to be used for the problem of discrimination of time series coming from 15 chaotic and non-chaotic models. Another issue related to analyzing the observation data of dynamical models is forecasting—future values are predicted on the basis of previous ones. In [25], classical approaches with feedforward neural networks as well as an LSTM architecture are investigated.

Due to the limited range of systems analyzed in previous studies, an extended dataset was developed in [14]—following the description from Prof. Sprott’s book [16]—to enable broader and more in-depth exploration of signal characteristics. This dataset includes signals from 15 dynamical systems—comprising five chaotic and ten non-chaotic ones—spanning first-order, second-order, and third-order models. To evaluate the dataset’s applicability, a series of experiments was performed using wavelet transforms as well as LSTM and CNN (Convolutional Neural Network) architectures for signal classification [17,26]. This study was further developed by introducing refined signals with similar initial conditions, generated from synthetic signals based on well-known chaotic and non-chaotic dynamical systems [15].

## 3. Materials and Methods

The work presented in this paper focuses on the effectiveness of deep learning models built on the refined signals for distinguishing between chaotic and non-chaotic behaviour under varying levels of noise.

### 3.1. Method for Obtaining Refined Signals

To support identification of chaotic systems based on result time series, a method of data reorganization was developed in [15]. Because of the property of chaotic systems consisting of their special sensitivity to initial conditions, it was proposed to generate short refined signals from long input signals, but with similar (not significantly different) initial conditions. This should help to distinguish between chaotic systems and non-chaotic ones.

**Figure 1:**
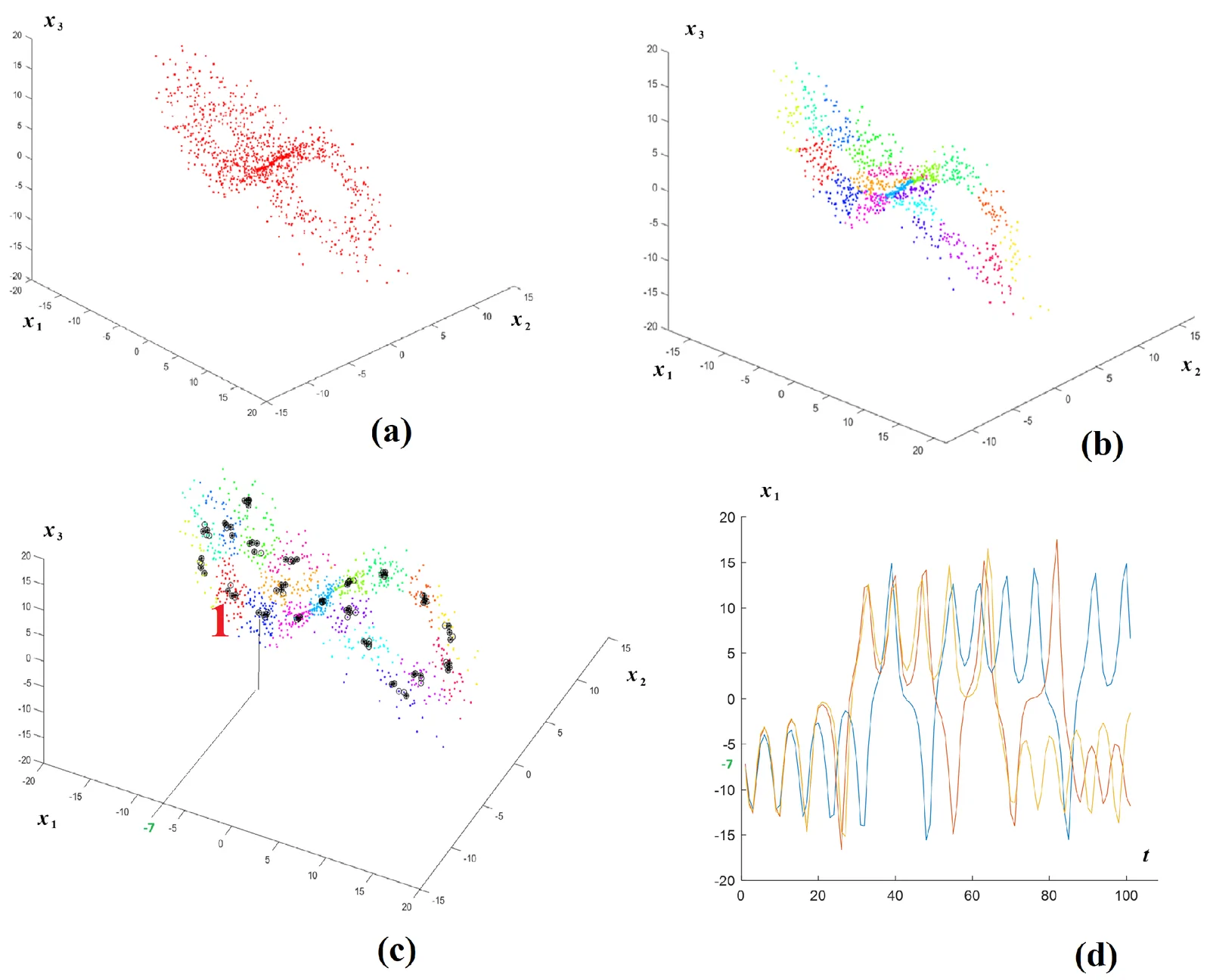
Obtaining refined signals for Lorenz system: (**a**) reconstructed 3D phase portrait ($x_{1}$—signal; $x_{2}$—the 1st derivative; $x_{3}$—the 2nd derivative) created from a long (1000 samples length) original input single signal, (**b**) clustered phase portrait (different clusters are marked with colors), (**c**) selected points (from a cluster no 1) which are close to its center but distant enough in time (min. 100 units), denoted by circles with asterisk, and (**d**) three short (100 samples each) refined signals with similar initial conditions taken from cluster no. 1 from (**c**).

In the first step, a multidimensional phase portrait is reconstructed based on a one-dimensional signal (the order of the model is well-known or is determined using the Takens’ Embedding method [27]). An example of such a reconstruction for a three-dimensional chaotic Lorenz system is shown in Figure 1a. The axes of the coordinate system are the state variables corresponding to the signal value, its derivative, and the second derivative.

In the second step, the phase-space points are clustered (Figure 1b), e.g., using the Fuzzy C-Means method [28,29]. In this step, the original sequences of length N=1000 were considered. For such sequences, the values of state variables (signal, the first derivative, the second derivative,…) were calculated and constitute an M-dimensional phase space. The dimension of the phase space depends on *M*—the order of the dynamic model analyzed. The phase space was clustered using the FCM method using the MATLAB R2025a (version 25.1) fcm function. The number of classes *C*, was based on the rule that the number of classes should be less or equal to $\sqrt{(}N)\approx 30$, and we assumed C=20. The fcm function was used with the following parameters: fuzzy partition matrix exponent m=2, minimum objective function improvement MinImprovement=1e−1,MaxNumIteration=100.

In the third step, the points (Figure 1c) closest to the cluster center (close in the sense of phase space) but possibly distant in the sense of moment of time are selected.

These point coordinates are the initial condition vectors for the resulting refined signals. Obtaining a refined signal consists of taking/cutting a subsequence from the input signal, starting from a given initial condition. Examples of three refined signals for cluster number 1 visible in Figure 1c, consisting of three separate points, are shown in Figure 1d.

### 3.2. Modeling Additive Noise for Signal Contamination

The signals (*x*) described above were contaminated using two different approaches. First, the signals were combined with Gaussian noise with mean m=0 and standard deviation σ=1 in selected experiments. Subsequently, Gaussian noise (*g*) with varying power levels (${SNR}_{dB}$) was used to obtain the resulting signal (*y*).

In the first noise condition, zero-mean Gaussian noise with a fixed standard deviation of σ=1 was added independently of the signal power. This condition was included as a fixed-variance noise stress test rather than as a common relative noise level. Consequently, its equivalent SNR varied across the considered systems, from approximately −5 to 18 dB, and the results obtained under this condition should not be interpreted as representing the same degree of signal degradation for all systems.

The following equations describe the individual stages of this process:

- The average signal power:
  (1) $$P_{\mathrm{signal}}=E\left[x^{2}\right]$$
  where E[·] denotes the expectation operator, and $E[x^{2}]$ is the mean-square signal power.
- The desired signal-to-noise ratio (SNR):
  (2) $$SNR_{\mathrm{lin}}=10^{\frac{SNR_{\mathrm{dB}}}{10}}.$$
- The required noise power:
  (3) $$P_{\mathrm{noise}}=\frac{P_{\mathrm{signal}}}{SNR_{\mathrm{lin}}}.$$
- Zero-mean Gaussian noise with variance equal to the calculated noise power:
  (4) $$g∼N(0,P_{\mathrm{noise}}).$$
- The noisy signal:
  (5) y=x+g.

### 3.3. Description of the Datasets

Four datasets were used in this study. The first of them included a group of signals from [14], hereafter referred to as the *Original* dataset, among which the following were considered:

1. Chaotic signals
  - CHA_1—Ueda system;
  - CHA_2—Lorenz system;
  - CHA_3—Rössler system.
2. Non-chaotic signals—linear continuous dynamical system
  - IOSC—rising oscillator;
  - DOSC_2—damped oscillator;
  - OSC_1—undamped oscillator.

The second set contained new signal representations generated using the transformation method described above, hereafter referred to as the *Refined* dataset, and consisted of the following:

1. Chaotic signals
  - C0ModelUedaA45—Ueda system;
  - C0ModelLorenzA51—Lorenz system;
  - C0ModelRoslerA52—Rössler system,
2. Non-chaotic signals—linear continuous dynamical system
  - C1LinearOscBooming01—rising oscillator;
  - C1LinearOscFading02—damped oscillator;
  - C1LinearOscillator01—undamped oscillator;
  - C2LinearFading01—linear dumped system.

Additionally, a third dataset, the *Augmented* one, was analyzed in this study, created by merging signals from (1) the *Original* dataset and (2) the *Refined* dataset.

Model validation was performed using the fourth dataset, hereafter referred to as the *Test* dataset, as follows:

1. Chaotic signals
  - CHA_4—Halvorsen system;
  - CHA_5—Rucklidge system.
2. Non-chaotic signals—linear continuous dynamical system
  - DOSC_1—damped oscillator;
  - OSC_2—undamped oscillator;
  - DS_2—damped system.

For each dataset, time series of length *l* (l=100), representing two classes (chaotic and non-chaotic), were extracted and used in the subsequent analysis. The number of samples for each dataset is reported in Table 1.

### 3.4. Experimental Setup and Model Architecture

Three different conditions were examined to assess the impact of using the *Refined* dataset on model performance and to compare the results with those obtained using the *Original* dataset. A recurrent LSTM network was evaluated under three scenarios:

- Training on noise-free *Refined*, *Original* and *Augmented* datasets and testing on noisy *Test* dataset;
- Training and testing on noise-contaminated datasets;
- Training on noise-contaminated *Refined* and *Original* datasets while testing on *Test* dataset characterized by different signal-to-noise ratios (SNRs).

This experimental design enabled a systematic assessment of the model’s performance and robustness under diverse noise conditions.

SNR levels of 2, 5, and 10 dB were selected to provide representative measurement conditions of low, moderate, and relatively high quality. They provide noise-to-signal power ratios of approximately 0.631, 0.316, and 0.1, respectively, thus enabling a controlled assessment of the classifier robustness with gradually increasing measurement uncertainty. Values of approximately 60%, 30%, and 10% were chosen arbitrarily.

The σ=1 condition represents fixed-variance noise and has a system-dependent equivalent SNR level ranging from about −5 to 18.

The LSTM network was selected to investigate whether a comparatively simple and well-established architecture can provide satisfactory results for the considered problem, while also serving as a baseline for the evaluation of more sophisticated models.

**Figure 2:**
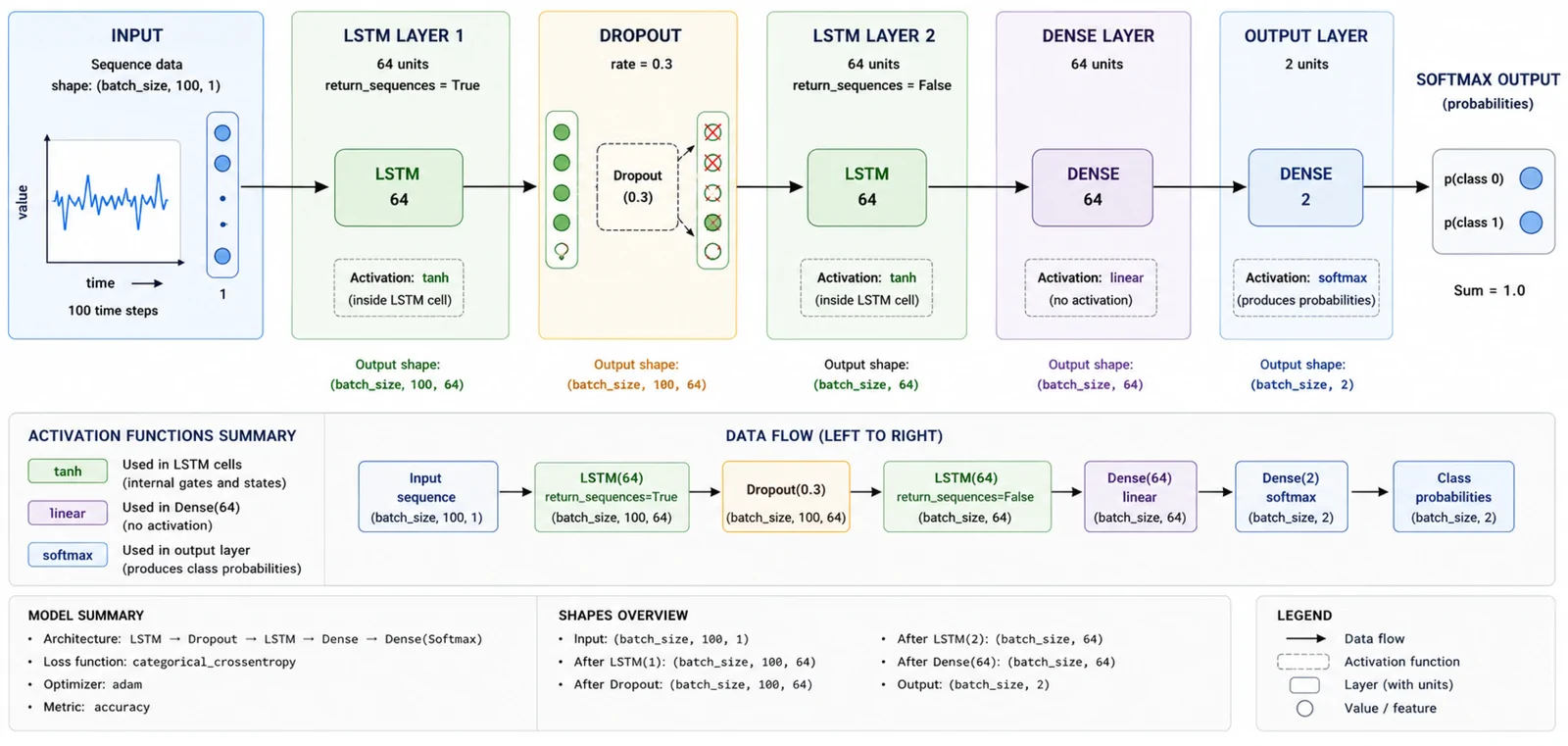
The LSTM model used in the experiments: two recurrent layers with 64 units, and a dropout layer with a rate of 0.3.

Previous studies ([14,15]) investigated various LSTM architectures, including single-layer and two-layer networks with different numbers of neurons. These experiments aimed to assess the impact of architectural choices on performance and to identify suitable configurations for further analysis. Based on these results, a two-layer LSTM architecture with *u* units (u=64), as shown in Figure 2, was selected for subsequent experiments.

Training was performed using the Adam optimizer with standard parameters (learning rate = 0.001) [30], while categorical cross-entropy was used as the loss function. All experiments were conducted on a computer equipped with an Intel(R) Core(TM) i9-10900F CPU @ 2.80 GHz and 64 GB of RAM, running a 64-bit operating system.

All models were trained for 250 epochs with a batch size of 64.

## 4. Experiments and Results

To investigate the influence of the training dataset and signal noise on model performance, three experimental scenarios were analyzed. Each scenario corresponds to a different combination of training datasets and signal-to-noise ratio (SNR) conditions during training and testing.

### 4.1. Scenario 1: LSTM Trained on Noise-Free Data, Tested on Noisy Data

Three LSTM models were trained independently on the *Refined*, *Original*, and *Augmented* datasets for comparison purposes. Their evaluation was conducted using a common *Test* dataset contaminated with varying levels of noise. Several metrics were used to assess model performance, including accuracy (Acc), Cohen’s Kappa coefficient (κ), true positive rate (TPR), true negative rate (TNR), macro-averaged F1-score (F1), and balanced accuracy (B_Acc).

In this study, TNR refers to correctly classified chaotic signals, while TPR refers to correctly classified non-chaotic signals, with non-chaotic signals defined as the positive class. To quantify the variability arising from stochastic model training, each model was trained 11 times using matched random seeds. The reported means and standard deviations therefore describe between-run variability for the same fixed training and test datasets. The reported results are averaged over all runs, and the standard deviation is provided in brackets.

Since the models trained on the *Original* and *Augmented* datasets yielded similar results, only the performance on the *Refined* and *Original* datasets is further analyzed and presented in Table 2.

Compared with models trained on the *Original* dataset, models trained on the *Refined* dataset achieved higher accuracy, Cohen’s κ, TPR, F1, and balanced accuracy, while TNR consistently decreased.

To assess whether the observed performance differences were consistent with respect to stochastic model training, paired Wilcoxon signed-rank tests were applied to the run-level metrics obtained from 11 matched random seeds. Because all models were evaluated using the same fixed datasets, the resulting *p*-values quantify consistency across training runs. In Table 3, exact two-sided *p*-values were provided, with the Holm correction applied to account for multiple comparisons across the six metrics within each noise condition. The rank-biserial correlation (RBC) was reported as a measure of effect size and calculated from paired differences between the *Refined* and *Original* datasets, such that positive values indicate higher performance for the *Refined* dataset, whereas negative values indicate higher performance for the *Original* dataset. The RBC confirmed the direction of these differences, with positive effects for accuracy, Cohen’s κ, TPR, F1, and balanced accuracy (RBC=1.00), and a negative effect for TNR (RBC=−1.00).

In particular, across the 11 matched training runs, the use of the *Refined* dataset resulted in higher average classification performance under all investigated noise conditions. The paired run-level comparisons showed consistently higher accuracy, Cohen’s κ, TPR, F1, and balanced accuracy for models trained on the *Refined* dataset (RBC=1.00, Holm $p_{Holm}=0.006$). The largest improvement was observed for TPR, which increased from 0.02 to 0.60 under σ=1, from 0.21 to 0.77 at ${SNR}_{dB}=2$, from 0.29 to 0.76 at ${SNR}_{dB}=5$, and from 0.47 to 0.84 at ${SNR}_{dB}=10$. In contrast, TNR was lower, with RBC=−1.00 and Holm $p_{Holm}=0.006$ across all noise conditions. Thus, the improvement associated with models trained on the *Refined* dataset was primarily driven by an increase in sensitivity (TPR corresponding to non-chaotic signals), accompanied by a moderate reduction in specificity (TNR corresponding to chaotic signals). Despite this trade-off, the overall classification performance remained consistently higher for models trained on the *Refined* dataset.

### 4.2. Scenario 2: LSTM Trained and Tested on Noisy Data

The subsequent experiments aimed to investigate whether and how model performance changes when trained on noisy data contaminated with noise having the same characteristics as those applied to the *Test* dataset. For this purpose, two datasets were used: the *Refined* and *Original* datasets. The same experimental conditions were maintained, and training was repeated 11 times using the same set of matched random seeds as in Scenario 1 (Section 4.1). Four noise characteristics were also analysed. The results obtained from these experiments are summarized in Table 4.

Statistical significance in the second experiment was assessed using the same procedure as in the previous experiment, including paired Wilcoxon signed-rank tests, Holm correction for multiple comparisons, and the rank-biserial correlation (RBC) as a measure of effect size. The statistical test results are presented in Table 5.

The paired run-level analysis indicated that the direction and consistency of the differences between models trained on the *Original* and *Refined* datasets depended strongly on the noise level. Under the fixed-variance noise (σ=1), statistically significant differences after Holm correction were observed only for TNR ($p_{\mathrm{Holm}}=0.012$), with a large negative effect (RBC=−0.97), indicating lower TNR for the *Refined* dataset. Although balanced accuracy showed a relatively large negative effect (RBC=−0.79), this difference did not remain statistically significant after correction ($p_{\mathrm{Holm}}=0.093$). The remaining metrics also showed no statistically significant differences after Holm correction.

For the higher noise intensity (${SNR}_{dB}=2$ and ${SNR}_{dB}=5$), the direction of the observed effects changed. At ${SNR}_{dB}=2$, accuracy, Cohen’s κ, TNR, F1, and balanced accuracy showed statistically significant positive effects (RBC: 0.94–0.97), indicating higher performance for the models trained on the *Refined* dataset, whereas TPR did not differ significantly (RBC=0.00, $p_{\mathrm{Holm}}=1.000$). At ${SNR}_{dB}=5$, the same metrics again showed significant positive effects (RBC: 0.91–0.97), while TPR showed a significant negative effect (RBC=−0.76, $p_{\mathrm{Holm}}=0.024$).

At ${SNR}_{dB}=10$, the direction of the effect was reversed for all metrics. Accuracy, Cohen’s κ, F1, and balanced accuracy showed significant negative effects: RBC=−0.97 and $p_{\mathrm{Holm}}=0.012$, while TPR: RBC=−0.76 and $p_{\mathrm{Holm}}=0.027$, and TNR: RBC=−0.82 and $p_{\mathrm{Holm}}=0.027$.

These findings indicate that the effect of the *Refined* dataset was dependent on the noise level applied to the training data, with the benefits of the refined representation varying according to the degree of training signal degradation.

### 4.3. Scenario 3: LSTM Trained and Tested on Noisy Data with Varying Signal-to-Noise Ratios

Finally, in an additional experiment, the signal-to-noise ratio was varied between the training and test sets, with the training set (*Refined* and *Original*) contaminated at ${SNR}_{dB}=2$ and the *Test* set at ${SNR}_{dB}=10$. As in the previous setups, this procedure was repeated 11 times using the same random seeds. The resulting metric values were averaged over all runs and are shown in Table 6, with standard deviations reported in brackets.

As presented in the table, the model trained on the *Refined* dataset outperformed that trained on the *Original* dataset in terms of accuracy, Cohen’s κ, TNR, F1, and balanced accuracy. Accuracy increased from 0.67 to 0.76, while Cohen’s κ increased from 0.24 to 0.51. The most pronounced improvement was observed for TNR, which increased from 0.40 to 0.77, indicating a substantial reduction in false-positive classifications. F1 and balanced accuracy also increased from 0.61 to 0.75 and from 0.61 to 0.76, respectively. In contrast, TPR decreased slightly from 0.83 to 0.75.

The paired Wilcoxon signed-rank tests showed that the differences between the two training conditions were consistent across the 11 matched training runs for all six evaluation metrics after Holm correction (Table 7). The rank-biserial correlations indicated strong effects for all metrics, with positive effects for accuracy, Cohen’s κ, TNR, F1, and balanced accuracy (RBC ranged from 0.88 to 1.00), and a negative effect for TPR (RBC=−1.00). These results indicate that the performance improvement associated with the *Refined* dataset was primarily driven by a substantial increase in specificity (TNR), which compensated for the moderate decrease in sensitivity (TPR). This improved balance between sensitivity and specificity resulted in higher F1 and balanced accuracy, as well as better overall classification performance under these noise conditions.

### 4.4. Comparison with Chaos Decision Tree Algorithm

In the 0–1 test, the analysed signal is transformed into auxiliary variables *p* and *q*, which form an artificial two-dimensional trajectory. Regular dynamics produce bounded motion, whereas chaotic dynamics produce diffusion-like motion. The statistic *K* quantifies this behaviour: K≈0 indicates regularity and K≈1 indicates chaos. However, noise can also induce diffusion and shift *K* towards one.

For the periodic transcription signal contaminated with 40% measurement noise, *K* remained close to one without denoising (0.997) for the raw signal and 0.982 after downsampling. After Schreiber denoising, downsampling reduced *K* from 0.991 to 0.364, bringing it closer to the range expected for periodic dynamics. Permutation entropy (PE) is fast and invariant under monotonic transformations, but noise alters the ordering of nearby observations and increases ordinal-pattern diversity; consequently, high PE may indicate either deterministic chaos or stochasticity, which is why Toker et al. [13] combined it with surrogate-data testing.

For the eight independent test cases not used during method development, the overall accuracy calculated from their results depended strongly on both signal length and measurement noise. For noise-free signals, it was 96.3% (770/800) at segment length 10,000 and 89.6% (717/800) at length 1000. When the standard deviation of the added measurement noise reached 40% of the signal standard deviation, the accuracy decreased to 83.9% (671/800) and 61.1% (489/800), respectively.

To assess whether this robustness generalizes to shorter signals, we applied the original *Chaos Decision Tree* implementation to the *Test* dataset. Two segment lengths, 100 and 1000 samples, were analysed under clean, Gaussian-noise (σ=1) and three SNR conditions. For binary evaluation, the *Chaos Decision Tree* output chaotic was mapped to the chaos class, whereas periodic and stochastic signals were jointly mapped to non-chaos. Results are presented in Table 8.

For noise-free data, increasing the segment length from 100 to 1000 samples improved chaos sensitivity from 27.90% to 91.40%. Under noise, however, performance collapsed: for 1000-sample segments, chaos sensitivity was 23.14% under σ=1 noise and only 4.88%, 1.22% and 0.11% at SNR values of 10, 5 and 2 dB. For 100-sample segments, it remained below 1% under all noisy conditions. The original implementation also exhibited numerical problems for some segments.

Although non-chaos sensitivity approached 100% under noise, this result was mainly caused by the classification of almost all signals as stochastic. At l=1000 and SNR 10 dB, 1792 of 1884 chaotic segments were classified as stochastic before the 0–1 test was reached; consequently, *K* was not calculated. Balanced accuracy was therefore only 52.44% and decreased to 50.02% at SNR 2 dB. These findings show that the stochasticity test, rather than the final 0–1 test, was the principal limitation for short noisy signals. Overall, the results indicate that the *Chaos Decision Tree* pipeline is effective primarily for sufficiently long, clean signals and that its reported noise robustness does not generalize reliably to short segments.

## 5. Discussion

The role of the FCM-based procedure is to select representative phase-space regions and signals associated with similar phase-space conditions. The LSTM is subsequently applied to analyze the temporal evolution of the resulting trajectories. Although the proposed procedure is motivated by differences in the dynamical evolution of the considered systems, including the sensitivity of chaotic systems to initial conditions, the clustering stage may also reflect differences in the underlying phase-space geometry. Therefore, the proposed framework should be interpreted as a combined phase-space selection and temporal classification approach.

The results obtained in this study indicate that the proposed transformation of the original signals has proven to be a valuable step in improving the classification of noise-contaminated signals. In experiments involving noise-free training data and noise-contaminated test data, models trained on the *Refined* dataset achieved consistently higher performance across the 11 matched training runs.

Based on the values reported in Table 2, the improvement in accuracy ranged from 17 to 31 percentage points, while the improvement in balanced accuracy ranged from 11 to 24 percentage points; the paired seed-level tests showed that these differences were consistent across the 11 training runs (see Table 3). This indicates that the *Refined* dataset provides more discriminative representations that remain robust under test noisy conditions. Moreover, although chaotic signals were easier to recognize than non-chaotic ones in both cases, as indicated by higher TNR values than TPR values, the difference in classification performance between the two classes was smaller for models trained on the *Refined* dataset. In contrast, substantially larger differences were observed when the *Original* dataset was used for model training.

These findings are further supported by the analysis of Cohen’s Kappa coefficient. For the *Original* dataset, κ values ranged from 0.00 to 0.40, indicating poor to moderate agreement, whereas the *Refined* dataset achieved values between 0.42 and 0.67, corresponding to moderate to good agreement.

The advantage of the LSTM model trained on the *Refined* dataset over the model trained on the *Original* dataset was also observed in Scenario 2 (Section 4.2), in which noise was added to the training data (see Table 4 and Table 5). Paired seed-level differences favouring the Refined dataset were observed consistently at ${SNR}_{dB}=2$ and ${SNR}_{dB}=5$. Under Gaussian noise with σ=1, the performance of models trained on the two datasets did not differ significantly, with the exception of TNR, which was found to be significantly higher for the *Original* dataset. In contrast, at ${SNR}_{dB}=10$, models trained on the *Original* dataset achieved better average performance. These results indicate that the advantage of the *Refined* dataset is particularly evident under moderate-to-high noise levels, but does not translate into a consistent improvement across all noise conditions.

It is worth noting that, in this group of experiments, the differences in performance between the models trained on the *Refined* and *Original* datasets became considerably smaller. This reduction in the performance gap was primarily associated with improved classification of non-chaotic signals by models trained on the *Original* dataset, as reflected by an increase in the true positive rate (TPR) for this class.

Finally, the model trained on the *Refined* dataset outperformed the model trained on the *Original* dataset in the final experiment, which involved applying different noise levels to both the training and test datasets. The *Refined* dataset yielded higher mean accuracy and balanced accuracy, with paired seed-level differences that were consistent across the 11 training runs. The reduction in the difference between TPR and TNR indicates more uniform class-wise performance (see Table 6 and Table 7). Nevertheless, the model trained on the *Original* dataset achieved a significantly higher TPR, showing that it was more effective in identifying non-chaotic instances.

Taken together, the results obtained for the fixed datasets considered in the three scenarios suggest that the advantage of the *Refined* dataset was primarily associated with a better balance between sensitivity and specificity. Under the noise conditions in which the *Refined* dataset outperformed the *Original* dataset, this translated into higher values of accuracy, Cohen’s κ, F1-score, and balanced accuracy reflecting more uniform classification performance across both classes.

## 6. Conclusions

The study presented in this paper investigated the classification of nonlinear system dynamics based on refined signal representations. The signals were processed using the method proposed in [15], which generates new signals characterised by similar initial conditions derived from the original data. Previous studies demonstrated that the resulting *Refined* dataset enables deep learning models to effectively differentiate between chaotic and non-chaotic dynamics.

The main objective of this work was to investigate the robustness of the proposed refined signal representation under noisy conditions. The results demonstrated that the refined representation generally improved classification performance and robustness in situations where models trained on clean signals were applied to noisy data. In contrast, when models were trained using noisy signals, the effectiveness of the refined representation became dependent on the level of signal degradation. Its advantages were most evident at low signal-to-noise ratios, while the original representation often achieved superior performance under less demanding noise conditions. These findings highlight that the effectiveness of the refined representation is influenced not only by the severity of signal degradation but also by the noise characteristics of the training data.

The results also demonstrate the potential of refined signal representations to improve the robustness of deep learning-based classification of nonlinear dynamics in the presence of noise. This is particularly relevant for applications involving biological signals, where measurements are inherently affected by noise and other sources of variability.

The robustness analysis presented in this study is limited to additive white Gaussian noise, which provides a controlled and reproducible framework for assessing the sensitivity of the LSTM classifier to progressively degraded signal quality. However, real-world and clinical recordings may contain temporally correlated noise and structured artifacts, such as baseline fluctuations, motion-related disturbances, electrode artifacts, or transient interference, which are not captured by the Gaussian noise model and may affect the classifier differently. Therefore, the reported results should be interpreted as robustness to additive Gaussian measurement noise rather than as comprehensive robustness to all clinically relevant signal corruptions; evaluation under structured and correlated artifacts will be an important direction for future work.

While the obtained results are encouraging, several limitations of the present study should be considered when interpreting the findings. Among them, the unequal sizes of the *Original* and *Refined* training datasets and the imbalance between chaotic and non-chaotic samples can be mentioned. Future work will investigate the impact of these factors through experiments with balanced dataset sizes, obtained either by subsampling the *Original* dataset or expanding the *Refined* dataset, and by applying class imbalance handling techniques, including class weighting.

Some limitations of the present study could be that the non-chaotic class is represented in the refined set exclusively by the selected linear dynamical systems (mainly linear oscillators). Therefore, the reported results are based not on all possible non-chaotic regimes. Future studies will also include nonlinear periodic and quasi-periodic dynamics, limit cycles, and other non-chaotic regimes in refined signals for deeper generalizability of the proposed framework.

In addition, the proposed refinement procedure will be extended to other signal types and validated using experimentally acquired biomedical signals, including gait, eye-movement, ECG, and EMG signals.

While the present study considered synthetic signals generated from well-known dynamical systems, an important next step will be to construct and evaluate refined representations directly from experimentally acquired biological signals.

## Figures and Tables

**Table 1:** Description of the datasets—number of time-series segments (TS) type.

	Dataset Type			
TS Type	*Refined*	*Original*	*Augmented*	*Test*
all	700	70,000	70,700	48,840
chaotic	300	30,000	30,300	18,840
non-chaotic	400	40,000	40,400	30,000

**Table 2:** Comparison of classification performance between the models trained on the noise-free *Original* and *Refined* datasets and evaluated on the *Test* dataset contaminated with noise of different power levels.

Noise Condition	Method	Acc	κ	TPR	TNR	F1	B_Acc
σ=1	*Original*	0.39 (0.01)	0.00 (0.02)	0.02 (0.01)	0.97 (0.02)	0.30 (0.01)	0.50 (0.01)
	*Refined*	0.70 (0.05)	0.42 (0.09)	0.60 (0.04)	0.85 (0.06)	0.70 (0.05)	0.73 (0.05)
${SNR}_{dB}=2$	*Original*	0.51 (0.04)	0.17 (0.05)	0.21 (0.06)	1.00 (0.00)	0.48 (0.05)	0.60 (0.03)
	*Refined*	0.82 (0.03)	0.64 (0.07)	0.77 (0.05)	0.91 (0.05)	0.82 (0.03)	0.84 (0.04)
${SNR}_{dB}=5$	*Original*	0.56 (0.04)	0.24 (0.05)	0.29 (0.06)	0.99 (0.01)	0.54 (0.04)	0.64 (0.03)
	*Refined*	0.80 (0.03)	0.59 (0.06)	0.76 (0.05)	0.86 (0.08)	0.79 (0.03)	0.81 (0.04)
${SNR}_{dB}=10$	*Original*	0.67 (0.03)	0.40 (0.07)	0.47 (0.03)	0.99 (0.06)	0.67 (0.03)	0.73 (0.04)
	*Refined*	0.84 (0.04)	0.67 (0.07)	0.84 (0.06)	0.84 (0.05)	0.83 (0.03)	0.84 (0.03)

**Table 3:** Results of the paired Wilcoxon signed-rank tests for the LSTM network trained on a noise-free training set and evaluated on the *Test* dataset contaminated with noise of different power. Values are reported as the test statistic (*W*), exact two-sided *p*-value, rank-biserial correlation (RBC), and Holm *p*-value ($p_{Holm}$).

Metric	σ = 1				SNR_*dB*_ = 2				SNR_*dB*_ = 5				SNR_*dB*_ = 10			
	W	$p_{\mathrm{exact}}$	RBC	$p_{\mathrm{Holm}}$	W	$p_{\mathrm{exact}}$	RBC	$p_{\mathrm{Holm}}$	W	$p_{\mathrm{exact}}$	RBC	$p_{\mathrm{Holm}}$	W	$p_{\mathrm{exact}}$	RBC	$p_{\mathrm{Holm}}$
**Acc**	0	0.001	1.00	0.006	0	0.001	1.00	0.006	0	0.001	1.00	0.006	0	0.001	1.00	0.006
** κ **	0	0.001	1.00	0.006	0	0.001	1.00	0.006	0	0.001	1.00	0.006	0	0.001	1.00	0.006
**TPR**	0	0.001	1.00	0.006	0	0.001	1.00	0.006	0	0.001	1.00	0.006	0	0.001	1.00	0.006
**TNR**	0	0.001	−1.00	0.006	0	0.001	−1.00	0.006	0	0.001	−1.00	0.006	0	0.001	−1.00	0.006
**F1**	0	0.001	1.00	0.006	0	0.001	1.00	0.006	0	0.001	1.00	0.006	0	0.001	1.00	0.006
**B_Acc**	0	0.001	1.00	0.006	0	0.001	1.00	0.006	0	0.001	1.00	0.006	0	0.001	1.00	0.006

**Table 4:** Comparison of classification performance between the models trained on the *Original* dataset and *Refined* dataset and evaluated on the *Test* dataset, all datasets contaminated with noise at the same power levels.

Noise Condition	Method	Acc	κ	TPR	TNR	F1	B_Acc
σ=1	*Original*	0.83 (0.01)	0.67 (0.02)	0.76 (0.00)	0.94 (0.02)	0.83 (0.01)	0.85 (0.01)
	*Refined*	0.81 (0.03)	0.61 (0.07)	0.77 (0.06)	0.86 (0.08)	0.80 (0.03)	0.83 (0.04)
${SNR}_{dB}=2$	*Original*	0.66 (0.01)	0.27 (0.03)	0.75 (0.01)	0.52 (0.04)	0.64 (0.02)	0.63 (0.02)
	*Refined*	0.76 (0.06)	0.50 (0.13)	0.75 (0.08)	0.78 (0.13)	0.75 (0.06)	0.76 (0.07)
${SNR}_{dB}=5$	*Original*	0.72 (0.05)	0.42 (0.11)	0.77 (0.02)	0.65 (0.12)	0.71 (0.05)	0.71 (0.06)
	*Refined*	0.79 (0.03)	0.58 (0.06)	0.75 (0.02)	0.86 (0.07)	0.79 (0.03)	0.81 (0.04)
${SNR}_{dB}=10$	*Original*	0.86 (0.02)	0.71 (0.03)	0.82 (0.02)	0.92 (0.03)	0.85 (0.02)	0.86 (0.02)
	*Refined*	0.80 (0.03)	0.60 (0.05)	0.78 (0.03)	0.84 (0.05)	0.80 (0.03)	0.82 (0.04)

**Table 5:** Results of the paired Wilcoxon signed-rank tests for the LSTM network trained on the contaminated training sets and evaluated on the contaminated *Test* dataset, all datasets with noise at different power levels. Values are reported as the Wilcoxon signed-rank test statistic (*W*), exact two-sided *p*-value, rank-biserial correlation (RBC), and Holm *p*-value ($p_{Holm}$).

Metric	σ = 1				SNR_*dB*_ = 2				SNR_*dB*_ = 5				SNR_*dB*_ = 10			
	W	$p_{\mathrm{exact}}$	RBC	$p_{\mathrm{Holm}}$	W	$p_{\mathrm{exact}}$	RBC	$p_{\mathrm{Holm}}$	W	$p_{\mathrm{exact}}$	RBC	$p_{\mathrm{Holm}}$	W	$p_{\mathrm{exact}}$	RBC	$p_{\mathrm{Holm}}$
**Acc**	16	0.147	−0.52	0.270	1	0.002	0.97	0.012	3	0.005	0.91	0.024	1	0.002	−0.97	0.012
** κ **	13	0.083	−0.61	0.270	1	0.002	0.97	0.012	3	0.005	0.91	0.024	1	0.002	−0.97	0.012
**TPR**	12	0.067	0.64	0.270	33	1.000	0.00	1.000	8	0.024	−0.76	0.024	8	0.024	−0.76	0.027
**TNR**	1	0.002	−0.97	0.012	2	0.003	0.94	0.012	1	0.002	0.97	0.012	6	0.014	−0.82	0.027
**F1**	14	0.102	−0.58	0.270	1	0.002	0.97	0.012	3	0.005	0.91	0.024	1	0.002	−0.97	0.012
**B_Acc**	7	0.019	−0.79	0.093	1	0.002	0.97	0.012	3	0.005	0.91	0.024	1	0.002	−0.97	0.012

**Table 6:** Results for the LSTM network trained on both datasets contaminated by noise at ${SNR}_{dB}=2$ and tested on data contaminated with noise at ${SNR}_{dB}=10$.

Dataset Used	Acc	κ	TPR	TNR	F1	B_Acc
*Original*	0.67 (0.06)	0.24 (0.16)	0.83 (0.02)	0.40 (0.16)	0.61 (0.09)	0.61 (0.08)
*Refined*	0.76 (0.04)	0.51 (0.08)	0.75 (0.04)	0.77 (0.07)	0.75 (0.04)	0.76 (0.04)

**Table 7:** Results of the paired Wilcoxon signed-rank tests for the LSTM models trained on the dataset contaminated with noise at ${SNR}_{dB}=2$ and tested on data contaminated with noise at ${SNR}_{dB}=10$. Values are reported as the Wilcoxon signed-rank test statistic (*W*), exact two-sided *p*-value, rank-biserial correlation (RBC), and Holm *p*-value ($p_{Holm}$).

Metric	*W*	$p_{\mathrm{exact}}$	RBC	$p_{\mathrm{Holm}}$
**Acc**	4	0.007	0.88	0.009
** κ **	2	0.003	0.94	0.009
**TPR**	0	0.001	−1.00	0.006
**TNR**	0	0.001	1.00	0.006
**F1**	2	0.003	0.94	0.009
**B_Acc**	1	0.002	0.97	0.008

**Table 8:** Classification performance for different signal lengths and noise conditions.

*l*	Noise	Chaos Sensitivity	Non-Chaos Sensitivity	Balanced Accuracy	Accuracy
100	Noise-free	27.90%	33.39%	30.65%	31.27%
100	σ=1	0.35%	99.99%	50.17%	61.55%
100	${\mathrm{SNR}}_{\mathrm{dB}}=10$	0.05%	98.29%	49.17%	60.39%
100	${\mathrm{SNR}}_{\mathrm{dB}}=5$	0.02%	99.50%	49.76%	61.12%
100	${\mathrm{SNR}}_{\mathrm{dB}}=2$	0.02%	99.88%	49.95%	61.36%
1000	Noise-free	91.40%	66.67%	79.03%	76.21%
1000	σ=1	23.14%	99.93%	61.54%	70.31%
1000	${\mathrm{SNR}}_{\mathrm{dB}}=10$	4.88%	100.00%	52.44%	63.31%
1000	${\mathrm{SNR}}_{\mathrm{dB}}=5$	1.22%	100.00%	50.61%	61.90%
1000	${\mathrm{SNR}}_{\mathrm{dB}}=2$	0.11%	99.93%	50.02%	61.43%

## Data Availability

The datasets used in this study are publicly available at: *Original* dataset: https://figshare.com/projects/Datasets_for_learning_of_unknown_characteristics_of_dynamical_systems/140275 (accessed on 30 August 2026); *Refined* dataset: https://figshare.com/projects/Refined_Data_Sets_for_Better_Chaos_Detection/206641 (accessed on 30 August 2026); and *Test* dataset: https://figshare.com/projects/Datasets_for_learning_of_unknown_characteristics_of_dynamical_systems/140275 (accessed on 30 August 2026). The source code required to reproduce the experiments to obtain the *Original* that was used in this paper is available in the GitLab repository: https://gitlab.com/draugustyn/signal-data (accessed on 30 August 2026).
